# Evaluation of Potential Thrombin Inhibitors from the White Mangrove (*Laguncularia racemosa* (L.) C.F. Gaertn.)

**DOI:** 10.3390/md13074505

**Published:** 2015-07-21

**Authors:** Caroline Fabri Bittencourt Rodrigues, Henrique Hessel Gaeta, Mariana Novo Belchor, Marcelo José Pena Ferreira, Marcus Vinícius Terashima Pinho, Daniela de Oliveira Toyama, Marcos Hikari Toyama

**Affiliations:** 1 Universidade Estadual Paulista Julio de Mesquita Filho, UNESP, Praça Infante Dom Henrique, s/n, 11330-900 São Vicente, SP, Brazil; E-Mails: cfabri3@gmail.com (C.F.B.R.); henriquehg@gmail.com (H.H.G.); belchor.mariana@gmail.com (M.N.B.); mvpinho@gmail.com (M.V.T.P.); 2 Departamento de Botânica, Instituto de Biociências, Universidade de São Paulo, Rua do Matão, 277, 05508-090 São Paulo, SP, Brazil; E-Mail: marcelopena@ib.usp.br; 3 Unicastelo—Universidade Camilo Castelo Branco, Campus São Paulo, Rua Carolina Fonseca, 584 Itaquera São Paulo-SP, 08230-030 São Paulo, SP, Brazil; E-Mail: gaveira@yahoo.com.br

**Keywords:** thrombin, *Laguncularia racemosa*, flavonoids, plasma coagulation

## Abstract

The aim of this work was to verify the effects of methanol (MeOH) and hydroalcoholic (HA) extracts and their respective partition phases obtained from white mangrove (*Laguncularia racemosa* (L.) C.F. Gaertn.) leaves on human thrombin activity. Among the extracts and phases tested, only the ethyl acetate and butanolic partitions significantly inhibited human thrombin activity and the coagulation of plasma in the presence of this enzyme. Chromatographic analyses of the thrombin samples incubated with these phases revealed that different compounds were able to interact with thrombin. The butanolic phase of the MeOH extract had the most potent inhibitory effects, reducing enzymatic activity and thrombin-induced plasma coagulation. Two glycosylated flavonoids in this partition were identified as the most potent inhibitors of human thrombin activity, namely quercetin-3-*O*-arabinoside (QAra) and quercetin-3-*O*-rhamnoside (Qn). Chromatographic analyses of thrombin samples incubated with these flavonoids demonstrated the chemical modification of this enzyme, suggesting that the MeOH extract contained other compounds that both induced structural changes in thrombin and diminished its activity. In this article, we show that despite the near absence of the medical use of mangrove compounds, this plant contains natural compounds with potential therapeutic applications.

## 1. Introduction

The enzyme thrombin is involved in the final coagulation cascade, in which it is responsible for the formation of fibrin clots and the conversion of fibrinogen into fibrin. Thrombin also activates other substrates, such as factors V, VIII, XI, and XIII [1]. This enzyme can mediate additional clotting events and enhance the efficiency of coagulation and peeling. It also activates platelets through the proteolytic cleavage of protease-activated receptors (PARs) to form platelet aggregates [2]. Thrombin is the primary physiological mediator of platelet accumulation and fibrin thrombus formation at sites of vascular injury [3]. Moreover, it is regarded as the central mediator of thrombogenesis, and its inhibition may disrupt thrombus formation and minimize lesion formation [4]. Thrombin has chemotactic activity toward monocytes [5], acts as an effective mitogen for lymphocytes and mesenchymal cells [6], and causes the production of platelet growth factors in the endothelium [7]. Therefore, the direct inhibition of thrombin can be an effective anticoagulant strategy [1].

Bleeding disorders related to thrombin activity are very common; however, anticoagulant drugs have many side effects. Currently, the two drugs that are most commonly used to treat these bleeding disorders are heparin and warfarin. Heparin can cause many adverse effects, such as osteoporosis, anaphylactic shock, and thrombocytopenia, and warfarin has teratogenic effects [8,9,10]. The discovery of new, plant-derived drugs has led to the isolation of many compounds that are used clinically and as prototypes for the synthesis of new medications. Forty percent of the currently available therapeutics used in medicine have been estimated to be derived from natural sources [11].

Mangrove plants show great potential for use in the discovery of bioactive compounds due to the abiotic factors and severe environmental conditions that impose adaptive pressures on these plants, requiring morphological and metabolic changes. These species produce carbohydrates, polyphenols, and other chemical compounds that function in the defense of these plants and promote their effective development in harsh environments [12]. Extracts and chemicals from mangrove plants are mainly used in folk medicine as insecticides, pesticides, astringents, or tonics for healing sores, dysentery, and fever. Therefore, the extraction of new compounds from mangroves in addition to those already known to the pharmacopoeia remains open to further investigation [13,14,15,16]. *Laguncularia racemosa* (L.) C.F. Gaertn. (Combretaceae) is a perennial plant known as the white mangrove, which, along with *Avicennia* sp. and *Rhizophora mangle*, is one of the most characteristic Brazilian mangrove species [17]. For this reason, *L. racemosa* is the focus of the present study.

The objective of this study was to verify the effects of methanolic (MeOH) and hydroalcoholic (HA) extracts from the leaves of *L. racemosa* and their respective partition phases on the enzymatic activity and structure of human thrombin (TH).

## 2. Results and Discussion

**Figure 1:**
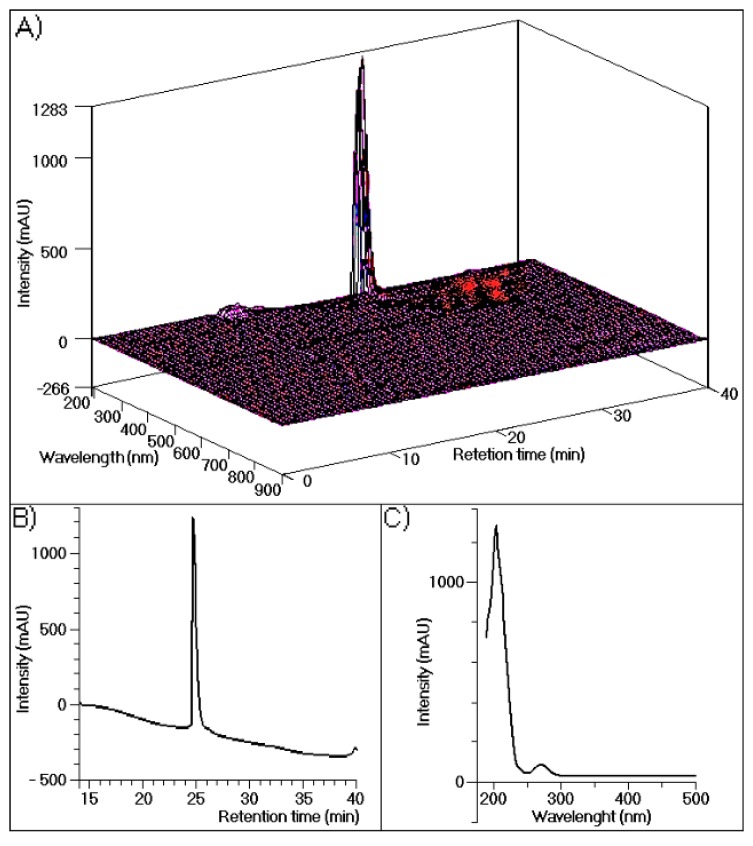
(**A**) The high-performance liquid chromatographic with a diode array detection, (HPLC-DAD) profile of human thrombin purified using a reverse-phase column (Discovery^®^ BioWide Pore C18, 25 cm × 10 mm, 10 μm). The sample was eluted with buffer A (0.1% trifluoroacetic acid (TFA)) and buffer B (66% acetonitrile (ACN) and 0.1% TFA) at a flow rate of 2 mL/min and the following gradient: 5 min, 100% buffer A; 30 min, 100% buffer B; and 36 min, 100% buffer A; (**B**) The HPLC profile of purified thrombin measured at 288 nm; (**C**) UV-Vis spectra of purified thrombin analyzed by performing UV scanning from 190 nm to 500 nm.

The results of chromatographic analyses of TH performed in this study are shown in Figure 1, which depicts three-dimensional UV absorption spectra data from 190 to 900 nm for each point along the chromatogram (Figure 1A). In this figure only one major peak corresponds to 95% of the whole fraction. Figure 1B shows the results of a simple analysis carried out at 280 nm, with only one visible protein peak. Figure 1C depicts the UV spectra of purified TH, with maximum absorption at approximately 200 nm and a second maximum absorption at 280 nm, demonstrating its purity.

**Figure 2:**
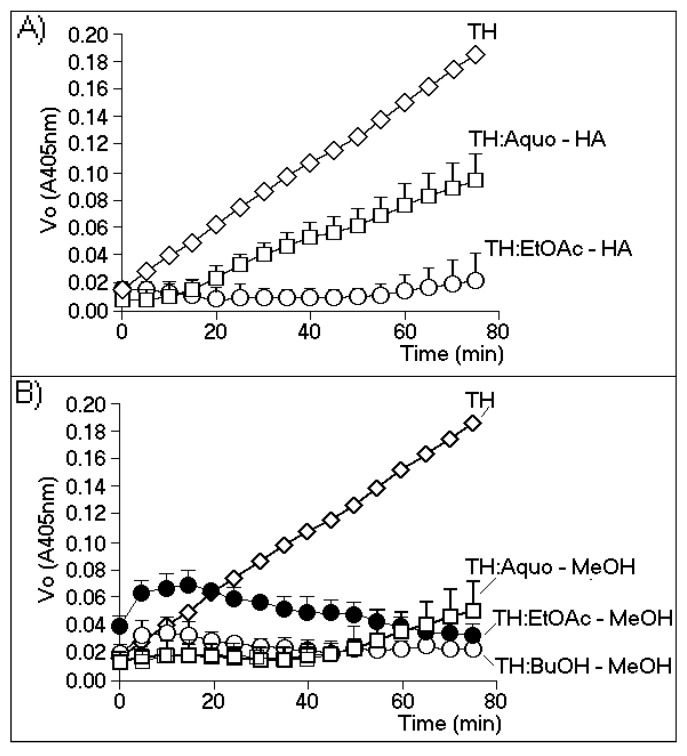
The chromogenic substrate for thrombin is specifically cleaved by thrombin at a slow rate. The biochemical reaction was b-Ala-Gly-Arg-*p*-nitroanilide → b-Ala-Gly-Arg + *p*-nitroanilide, which was measured at 405 nm. The method used for this evaluation was based on [18] and the manufacturer’s instructions. All enzymatic assays were performed using a SPECTRA MAX (Molecular Devices, Sunnyvale, CA, USA), with *n* = 12. (**A**) The effects of the aqueous phase (Aquo-HA) and ethyl acetate (EtOAc-HA) phase of the hydroalcoholic extract; (**B**) the inhibitory effects of the aqueous (Aquo-MeOH), ethyl acetate (EtOAc-MeOH) and butanolic (BuOH-MeOH) phases of the methanolic extract.

Figure 2A,B show the effects of the most effective phases of the HA and MeOH extracts, respectively. Figure 2A shows that the enzymatic activity of TH strongly decreased only when the thrombin samples were incubated with the ethyl acetate phase of the HA extract (EtOAc-HA). The aqueous phase (Aquo-HA) showed only a moderate inhibitory effect. Figure 2B shows that the aqueous and butanolic phases of the MeOH extract (Aquo-MeOH and BuOH-MeOH, respectively) possessed the highest inhibitory effects, although the observed differences between the two phases were not statistically significant. In addition, the inhibitory potential exhibited by the EtOAc-MeOH phase was likely due to the minor compounds present in this fraction. During the first interval (0–20 min) of the time course of the experiment, this phase showed a significant increase in the initial rate of enzymatic activity, whereas after this interval (from 20 min to 80 min), gradual inhibition of the enzyme was observed. Thus, the results obtained with the EtOAc-MeOH partition indicated the possible presence of both a thrombin inhibitor and activator. The treatment of TH with Aquo-MeOH resulted in the identification of two active components: one inhibitory component that represented the major and predominant group (0–50 min), and a second component appearing after 50 min that caused increased TH activity and was likely driven by the presence of a minor group of compounds. However, these results were not significant when subjected to statistical analysis.

Only the BuOH-MeOH phase showed predominantly homogeneous results, demonstrating an inhibitory effect on thrombin activity. Various substrates can be used to measure the thrombin activity of thrombin, but their use is limited by the rate of thrombin-mediated catalysis. Thus, the use of a chromogenic substrate for thrombin (Sigma Aldrich, Tokyo, Japan) allowed for the gradual evaluation of enzymatic activity, which enabled us to more effectively assess the influences of the different phases on the activity of this enzyme.

**Figure 3:**
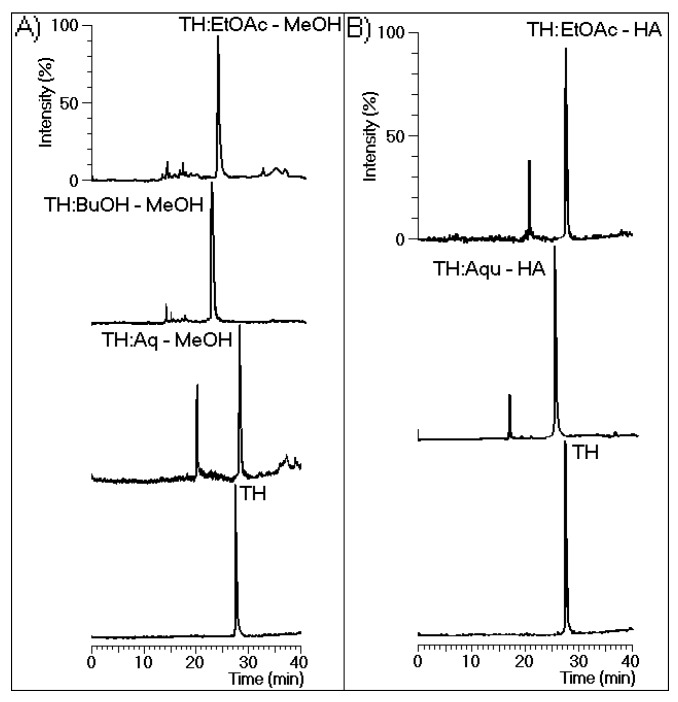
(**A**) The chromatographic profiles of thrombin (TH) before and after incubation with the aqueous (Aq-MeOH), butanolic (BuOH-MeOH), and ethyl acetate (EtOAc-MeOH) phases of the methanolic extract; (**B**) The chromatographic profiles of TH before and after incubation with the aqueous (Aqu-HA) and ethyl acetate (EtOAc-HA) extracts. All analyses were performed under the same chromatographic conditions used to evaluate the purity of TH presented in Figure 1. In this case, fluorescence was detected via chromatography at an excitation of 280 nm and an emission of 348 nm to specifically monitor the intrinsic fluorescence of tryptophan.

**Figure 4:**
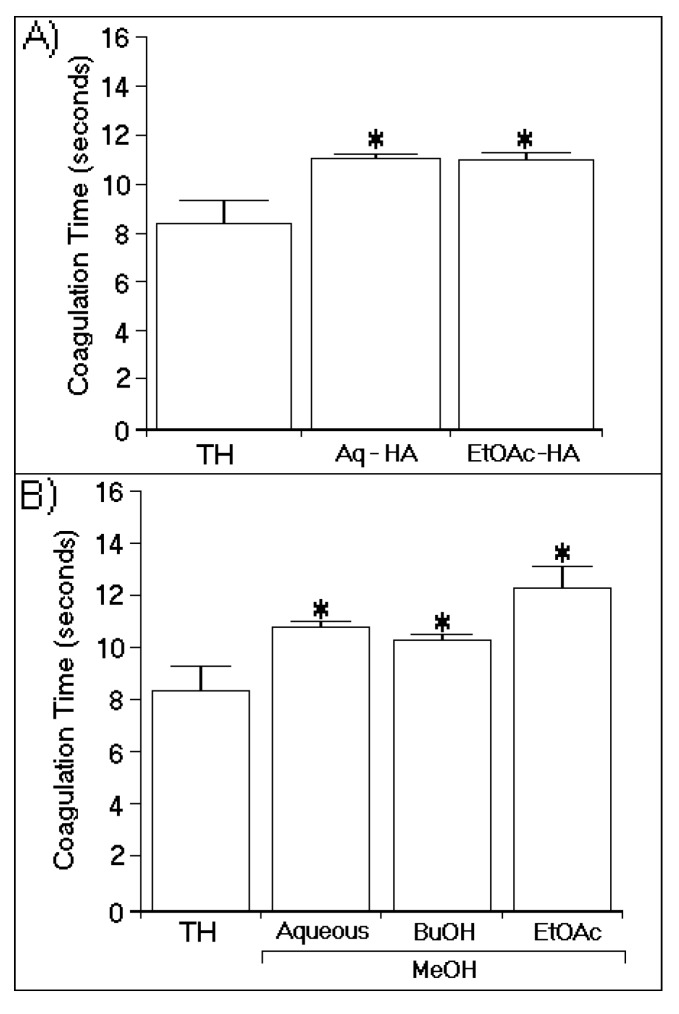
Effects of all phases of the hydroalcoholic (**A**) and methanolic (**B**) extracts on the coagulation time, which was measured using a turbidity test based on the average thrombin time. The bars represent the standard deviations, and * indicates significant differences relative to a standard. All analyses were performed using analysis of variance (ANOVA, *p* < 0.05), and each bar represents *n* = 12.

Figure 3A shows the chromatographic analysis results for the thrombin samples incubated with EtOAc-MeOH, BuOH-MeOH, and Aquo-MeOH. These analyses revealed that all MeOH phases induced changes in the retention time of the native protein. Only major chemical modifications to the protein were detected in all samples except for that treated with Aquo-MeOH. Figure 3B shows the high-performance liquid chromatography (HPLC) analysis results for the thrombin samples incubated with the EtOAc-HA and Aquo-HA phases, which both exhibited two main peaks. Figure 4 shows the effects of the EtOAc-MeOH, BuOH-MeOH, Aquo-MeOH, EtOAc-HA, and Aquo-HA phases on the coagulation time induced by native TH. According to the obtained results, all phases of the HA (Figure 4A) and MeOH (Figure 4B) extracts either diminished or partly inhibited thrombin activity. These samples also significantly increased the thrombin-induced clotting of citrated plasma. The most potent phase was EtOAc-MeOH.

**Figure 5:**
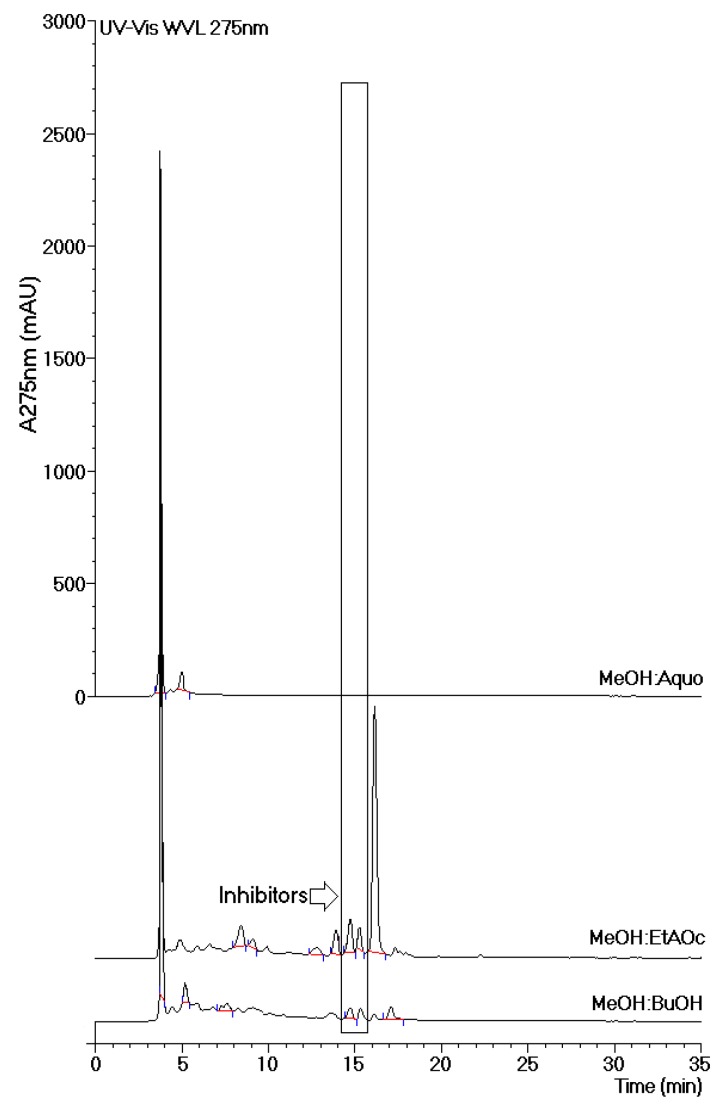
Chromatographic analyses of the aqueous (Aq-MeOH), butanolic (BuOH-MeOH), and ethyl acetate (EtOAc-MeOH) phases of the methanolic extract. The chromatographs were obtained by reverse-phase analysis. The most effective inhibition of thrombin activity and the greatest increase in the coagulation time were observed mainly in the BuOH-MeOH and EtOAc-MeOH phases of the methanolic extract. These results suggest that the main compounds responsible for these effects can be considered inhibitors.

Figure 5 shows the chromatographic profiles of all tested phases of the MeOH extract. Analysis of these chromatograms revealed the presence of two common peaks that were eluted at approximately 15 min. These peaks appeared in both phases (EtOAc-MeOH and BuOH-MeOH) of the MeOH extract, which exhibited the most effective inhibition of thrombin activity and showed an increase in the plasma coagulation time. Thus, the compounds present in both of these peaks were considered to be inhibitors.

The results obtained from enzymatic activity analysis showed significant differences in the inhibition profiles between the HA and MeOH extracts. The MeOH extract contained inhibitors as well as compounds with thrombin-promoting activities. In the HA extract, by contrast, only inhibitory compounds were observed. The MeOH extract had a significantly higher inhibitory capacity than the HA extract. In this study, we determined the chemical structures of two flavonoids identified in the EtOAc and BuOH phases of the MeOH extract. The isolation and evaluation of these flavonoids from the BuOH phase of the MeOH extract was the focus of this study. Our analysis showed the presence of at least two potential groups of inhibitors, one in the aqueous phase and the other in the BuOH phase, as indicated in Figure 5. The results of analyses depicted in Figure 2A and Figure 5 strongly suggest that the inhibitor found in the Aquo-MeOH phase might be chemically unstable, as the inhibitory potency of this compound was found to decrease over time.

For chromatographic analyses, TH samples were incubated with different phases of the MeOH or HA extracts. These samples exhibited one or two chemically modified thrombin peaks in the chromatograms. In addition, chromatographic analyses revealed that all of the peaks derived from the chemical modification of thrombin had different retention times. Moreover, these peaks all eluted with different retention times compared with that of native TH. We also showed that the *L. racemosa* leaves contain several other compounds with direct effects on TH, some of which can be considered as either enhancers or inhibitors of the activity of this enzyme.

A repeat assay testing the abilities of the different extracts to inhibit thrombin activity also showed that the BuOH-MeOH and EtOAc-HA phases had possible anticoagulant activities. This inhibition was likely associated with structural changes to the protein, as evidenced by the chromatographs of thrombin after incubation with the extracts. These data showed a change in the departure time of the protein, suggesting that a change in its activity occurred, likely due to an alteration in its catalytic site or allosteric regions.

Figure 4 shows that EtOAc-MeOH was the most effective phase of the MeOH extract and that it promoted a significant increase in the coagulation time. However, as shown in Figure 3, this phase showed bi-functional activity, acting as an enhancer and inhibitor of thrombin. Thus, the absence of these types of studies on white mangrove (*L. racemosa*) may be due to the fact that several compounds with antagonistic effects against thrombin are present, resulting in only marginal effects. However, some compounds with high potencies against thrombin were identified in this phase. Thus, this species may represent an alternative source of direct inhibitors of thrombin.

The most effective phases against thrombin were obtained from the MeOH extract. The chromatographic studies presented in Figure 5 show that all phases had an initial peak that eluted at 3.7 min. This peak was characterized as tannin by routine chemical assay. Tannins are common in mangroves and represent the most abundant group of highly antioxidative compounds present in these plants [19]. Our results led us to conclude that this group of tannins found in *L. racemosa* did not influence the plasma coagulation time or thrombin activity. HPLC analysis of Aquo-MeOH showed the presence of the tannin constituent as well as minor compounds that were likely responsible for the inhibition of thrombin activity and the increase in the plasma coagulation time. HPLC analyses of the EtOAc-MeOH and BuOH-MeOH phases showed common peaks that were designated as thrombin inhibitors. The relevant compounds were isolated from the BuOH-MeOH phase and were identified as quercetin-3-*O*-rhamnoside (Qn) and quercetin-3-*O*-arabinoside (QAra). These structures were identified using detailed mass spectroscopy and NMR experiments, and the resulting structures were compared to those of authentic samples and references.

**Figure 6:**
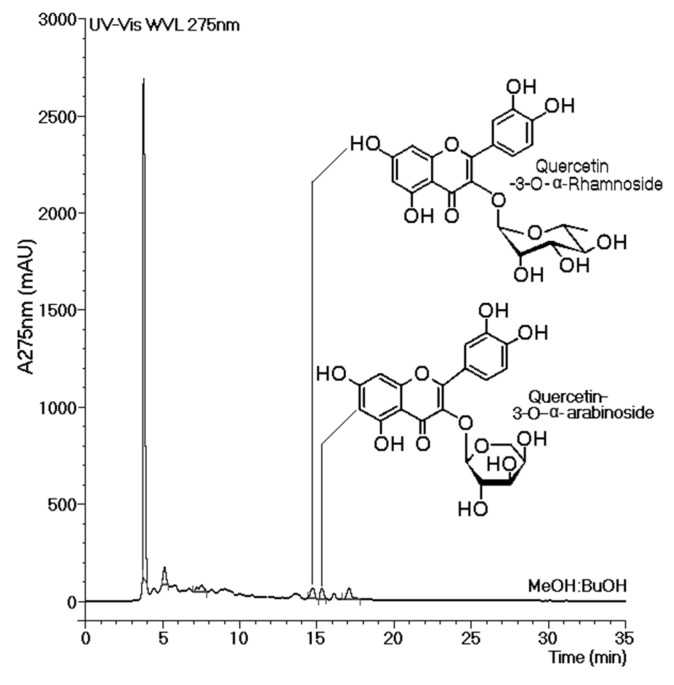
The chemical structures of quercetin-3-*O*-rhamnoside (Qn) and quercetin-3-*O*-arabinoside (QAra) obtained from the butanolic (BuOH-MeOH) phase of the methanolic extract.

**Figure 7:**
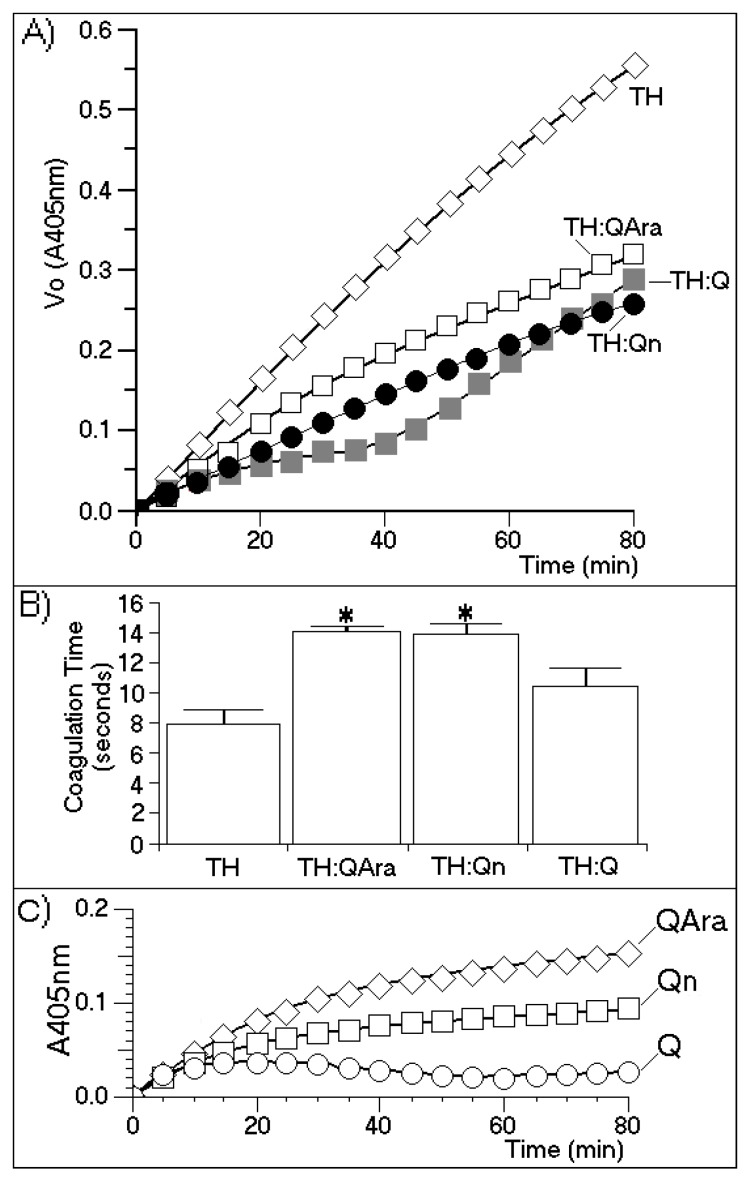
(**A**) A chromogenic substrate for thrombin was used to measure thrombin inhibition by quercetin-3-*O*-rhamnoside (Qn) and quercetin-3-*O*-arabinoside (QAra). This assay was performed under the same conditions described elsewhere in this paper; (**B**) The effects of Qn and QAra on the coagulation time were measured using a turbidity test based on the average thrombin time. The bars represent the standard deviation, and * indicates significant differences in relation to the standard. All analyses were performed using analysis of variance (ANOVA, *p* < 0.05), and each bar represents *n* = 12. The spectroscopy results varied for all purified flavonoids over the course of the enzymatic experiment; (**C**) Enzyme kinetics of the compounds with the chromogenic substrate to see if there was interaction between them.

**Figure 8:**
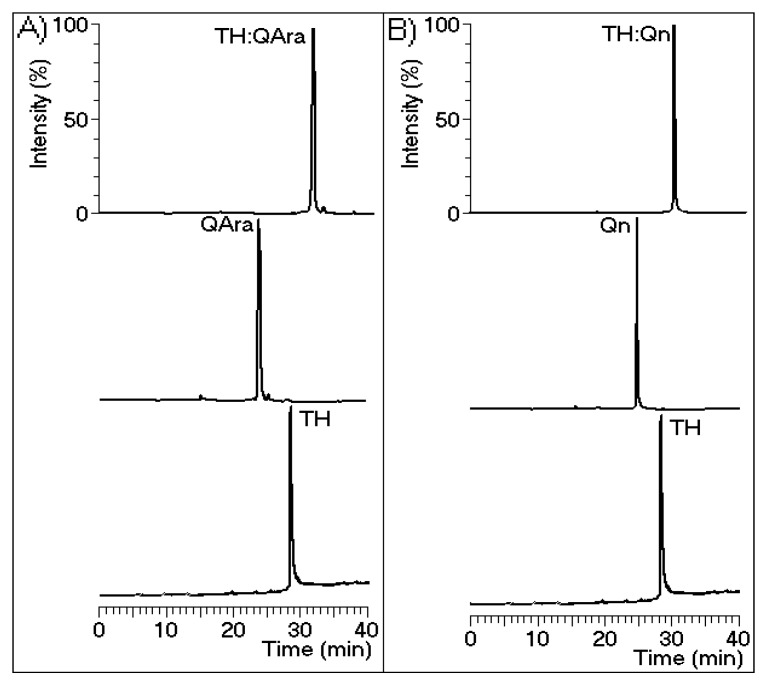
Chromatograms were generated using a reverse-phase C18 column with buffer A (trifluoroacetic acid (TFA) 0.1%) and buffer B (acetonitrile (ACN) 66% and TFA 0.1%) and detection at 280 nm. Isolated thrombin was incubated with the flavonoids prior to the chromatographic runs. There is a notable difference in the output timing of the protein. Figure (**A**) and (**B**) depict quercetin-3-*O*-arabinoside and quercetin-3-*O*-rhamnoside, respectively.

Figure 6 shows the molecular structures of both flavonoids purified from the BuOH-MeOH phase. These compounds were characterized by HPLC-mass spectrometry (HPLC-MS/MS) as Qn and QAra. Both of these flavonoids strongly diminished the activity of TH and its capacity to induce plasma coagulation. The results presented in Figure 7 show that the presence of carbohydrates induced a stable inhibition profile that was not observed for quercetin aglycone. Therefore, the sugars rhamnoside and arabinoside were clearly associated with the differences in the inhibitory potencies of these quercetin derivatives isolated from the white mangrove (*L. racemosa*). The final results showing the strong interactions of Qn and QAra with purified TH are shown in Figure 8. Under identical chromatographic conditions, native TH was eluted at 28.1 min, thrombin treated with Qn was eluted at 23.8 min, and thrombin treated with QAra was eluted at 25.2 min. Thus, both of these flavonoids significantly decreased the hydrophobicity of TH. 

The results presented in Figure 7 indicate that the sugars were involved in promoting the molecular stability of the interactions between the flavonoids and thrombin. These results indicated that Q almost completely inhibited TH activity for at least 30 min of the time course assay. After this time, a strong increase in TH activity was observed. These results were compared with the effects induced by Qn and QAra, which caused a stable decrease in thrombin activity for 80 min during the time course experiment. At 80 min, the maximum inhibitory effects of both glycosylated flavonoids were observed. However, quercetin also caused an increase in enzymatic activity. Figure 7B shows that the coagulation times increased for TH:QAra (interaction of thrombin with QAra), TH:Qn (thrombin with quercetin-3-*O*-rhamnoside) and TH:Q (thrombin with quercetin) compared with that of thrombin in the absence of flavonoids.

Some previous reports have shown that quercetin and other aglycone flavonoids reduce blood coagulation by inhibiting platelet aggregation induced by thrombin, ADP (adenosine diphosphate) or collagen. This inhibitory effect is similar to that exerted by phosphodiesterase inhibitor IBMX (3-isobutyl-1-methylxanthine) and dipyridamole [20]. Other studies have shown that quercetin and resveratrol from red wine inhibit thrombin activity and thus, platelet aggregation [21,22]. In addition, Bijak *et al.* [23] have shown that quercetin is the most potent inhibitor of the activity of this enzyme. Thus, our findings show that the structure of quercetin is important for its initial interaction with thrombin. According to our results, this complex dissociated after several minutes. As shown in Figure 7, the complex formed between thrombin and quercetin appeared unstable compared with those formed with either Qn or QAra. These results are novel and unexpected because some studies have reported that quercetin and myricetin are the optimal flavonoids for strong inhibition of thrombin activity [24]. The study conducted by Mozzicafreddo *et al.* [25] used conventional short-term enzymatic activity assays. However, our enzymatic activity assay involved the use of a chromogenic substrate for thrombin (beta-Ala-Gly-Arg *para*-nitroanilide), which is proteolytically cleaved by TH to produce beta-Ala-Gly-Arg and *p*-nitroanilide. These end-products were measured in our experiments according to the user manual. This method was specifically performed to assess anti-thrombin activity [18] because the thrombin generation chromogenic substrate is specifically cleaved by thrombin at a slow rate, which allows for an extension of the time course interval beyond that which can be used with a conventional substrate. This characteristic appears to be very important for observing the effects of potential TH inhibitors. Moreover, the enzymatic activity, plasma clotting time and HPLC analyses showed that Qn and QAra may be considered novel thrombin inhibitors obtained from *L. racemosa*. Both of these compounds were shown to interact with thrombin (Figure 8), diminishing its catalytic activity toward its substrates. The activities of these compounds contrast with those of heparin and its derivatives, which inhibit thrombin and other coagulation serine proteases [26].

## 3. Experimental Section

### 3.1. Reagents and Plant Materials

Pre-purified and standardized TH was purchased from Sigma-Aldrich. *Laguncularia racemosa* (L.) C.F. Gaertn. leaves were collected from the mangrove forest of Praia Grande (23°59′6.12″ S, 46°23′53.86″ W) in May 2012 and conditioned in plastic bags for transport to the Molecular Biology and Peptides Laboratory (BIOMOLPEP). The plant materials were cleaned, washed with distilled water, frozen in liquid nitrogen, and lyophilized for subsequent extraction. A voucher specimen was deposited at Herbário UNISANTA (HUSC) by Mariana Novo Belchor and Caroline Fabri Bittencourt Rodrigues under reference number 8256.

### 3.2. Extraction

Dried and powdered *L. racemosa* (39.4 g) leaves were defatted with hexane to obtain 19.4 g of crude hexane extract after removal of the solvent. The plant materials resulting from this first extraction were divided into two parts (portions 1 and 2), which were exhaustively extracted at room temperature with MeOH and an HA solution (4:6 water:MeOH), respectively, to obtain crude MeOH and HA extracts after removal of the solvent. Both crude extracts were resuspended in MeOH:water (1:4) and successively partitioned with hexane, dichloromethane (CH_2_Cl_2_), EtOAc and BuOH. The remaining aqueous phase was subjected to lyophilization. These processes resulted in the following masses for each phase: (1) crude MeOH extract: hexane phase (Hex-MeOH)—203 mg; CH_2_Cl_2_ phase (DCM-MeOH)—22 mg; EtOAc-MeOH—194 mg; BuOH-MeOH—372 mg; and Aquo-MeOH—1.22 g; (2) crude HA extract: Hex-HA—0.8 mg; DCM-HA—6.5 mg; and EtOAc-HA—330 mg. Both the BuOH-HA and Aquo-HA phases had low masses. Thus, only the polar extracts were used in this study.

### 3.3. HPLC Analysis

The thrombin-treated samples were subjected to spectroscopic analyses (UV analysis at 190–900 nm) and fluorescence-mediated chromatographic techniques by reverse-phase HPLC (Discovery^®^ BioWide Pore C18, 25 cm × 10 mm, 10 μm). These procedures were important for verifying the structural changes and formation of complexes between thrombin and the compounds extracted from *L. racemosa*. The data were analyzed using an FP-2020 scanning fluorescence detector (Jasco, Tokyo, Japan). UV data were acquired using an MD-2018 detector (Jasco). Running buffer A contained 0.1% trifluoroacetic acid (TFA), and buffer B included 66% acetonitrile and 0.1% TFA. The gradient was as follows at a flow rate of 2 mL/min for a total of 40 min: 5 min, 100% buffer A; 30 min, 100% buffer B; and 36 min, 100% buffer A. Fluorescence was monitored at an excitation wavelength of 280 nm and an emission wavelength of 348 nm with a photodiode array (PDA) at 280 nm and maximum absorbance ranging from 200 nm to 500 nm. 

### 3.4. Incubation of Thrombin with the Extracts and Their Respective Phases

Only the polar extracts (MeOH and HA) and their polar phases (EtOAc-MeOH, BuOH-MeOH, Aquo-MeOH, EtOAc-HA, and Aquo-HA) were used in thrombin assays. All samples were solubilized in dimethylsulfoxide (DMSO), and 10 µL of these solutions was added to 990 mL of saline.

### 3.5. Thrombin Enzymatic Assay

The potential inhibitory activities of the extracts against thrombin were also observed. The enzyme was dissolved in a 0.9% NaCl saline solution at a final concentration of 1 mg/mL. TH samples were incubated with the extracts in microplates for 20 min at room temperature in the presence of a chromogenic substrate for thrombin. The substrate was solubilized in water containing 50 mM Tris-HCl (pH 7.4) and 100 mM NaCl at 37 °C. A blank control (Bk = substrate + buffer + saline) and a positive control were also analyzed. The samples were read in sequential intervals (5 min, for a total time of 90 min) using a spectrophotometer (λ = 405 nm). For highly pure compounds, a concentration of 0.5 µmol/mL of compound that was previously incubated with TH was used.

### 3.6. Thrombin Time

The thrombin time test measures the time to clot formation mediated by thrombin in plasma using a turbidimeter (HumanClot, Wesbaden, Germany). To conduct this test, we used 25 µL of human plasma (P9523-5ML, Sigma-Aldrich), which was pre-incubated with the extracts and phases for 1 min at 37 °C. The clotting time was measured after the addition of 25 µL TH. Measurements were performed in triplicate. For highly pure compounds, a concentration of 0.5 µmol/mL of compound was used. Data were expressed as the mean ± standard deviation for *n* animals. Data were analyzed by analysis of variance (ANOVA) followed by *a posteriori* Dunnett’s testing. *p* Values of <0.05 were considered significant. 

### 3.7. Isolation and Structural Characterization of Glycosylated Flavonoids

The flavonoids were identified in the EtOAc and BuOH phases of the MeOH extract by HPLC-MS/MS. The chromatographic peaks at 14.8 min and 15.1 min presented *m*/*z* 433 and *m*/*z* 447, respectively, with base peaks in the negative ionization mode mass spectra and UV spectra characteristic of glycosylflavonoids (UV max: λ 227, 262, and 355 nm). Taken together, these data indicated the binding of quercetin derivatives to different sugars. The product ion spectra obtained from the negative ion MS/MS for the precursor ions (*m*/*z* 433 and *m*/*z* 447) were the same and both compounds showed *m*/*z* 301 as the base peak. Therefore, the presence of quercetin aglycone in their structures and the losses of their arabinosyl and rhamnosyl moieties, respectively, were confirmed. Thus, the structures were designated as QAra and Qn.

These compounds were isolated from the BuOH-MeOH phase of crude MeOH extract by semi-preparative HPLC. The following elution gradient was employed at a flow rate of 4.1 mL/min: solvent A = aqueous acetic acid, 0.1% (v/v); solvent B = MeOH; elution profile: 0–35 min, 30%–100% B (linear gradient). A UV-DAD detector was set to record between 210 and 600 nm, and UV chromatograms were recorded at 275 nm. The structures of both isolated compounds were examined by NMR.

Quercetin-3-*O*-arabinoside: ^1^H-NMR (300 MHz, CD_3_OD) δ 6.41 (d, *J* = 2.1 Hz, H-6), δ 6.23 (d, *J* = 2.1 Hz, H-8), δ 7.51 (d, *J* = 2.0 Hz, H-2′), δ 6.85 (d, *J* = 8.3 Hz, H-5′), δ 7.25 (dd, *J* = 8.3 Hz and 2.0 Hz, H-6′), δ 5.27 (d, *J* = 5.1 Hz, H-1″), δ 3.23–3.62 (H-2″–H-5″); ^13^C NMR (75 MHz, CD_3_OD) 156.1 (C-2), 133.5 (C-3), 177.3 (C-4), 161.1 (C-5), 98.5 (C-6), 164.3 (C-7), 93.4 (C-8), 155.9 (C-9), 103.5 (C-10), 120.3 (C-1′), 115.3 (C-2′), 145.3 (C-3′), 148.7 (C-4′), 115.7 (C-5′), 123.0 (C-6′), 100.9 (C-1″), 70.5 (C-2″), 71.4 (C-3″), 65.5 (C-4″), 63.6 (C-5″) [27,28].

Quercetin-3-*O*-rhamnoside (Quercitrin): ^1^H-NMR (300 MHz, CD_3_OD) δ 6.37 (d, *J* = 2.1 Hz, H-6), δ 6.19 (d, *J* = 2.1 Hz, H-8), δ 7.34 (d, *J* = 2.1 Hz, H-2′), δ 6.94 (d, *J* = 8.7 Hz, H-5′), δ 7.30 (dd, *J* = 8.4 Hz and 2.1 Hz, H-6′), δ 5.34 (d, *J* = 1.5 Hz, H-1″), δ 4.23 (dd, *J* = 3.0 Hz and 1.8 Hz, H-2″), δ 3.75 (dd, *J* = 9.0 Hz and 3.3 Hz, H-3″), δ 3.32 (dd, *J* = 9.3 Hz and 3.3 Hz, H-4″), δ 3.41 (d, *J* = 6.0 Hz, H-5″), δ 0.94 (d, *J* = 6.0 Hz, H-6″); ^13^C NMR (75 MHz, CD_3_OD) 159.2 (C-2), 136.5 (C-3), 179.7 (C-4), 163.1 (C-5), 99.8 (C-6), 165.8 (C-7), 94.9 (C-8), 158.8 (C-9), 106.2 (C-10), 123.1 (C-1′), 117.3 (C-2′), 146.7 (C-3′), 149.8 (C-4′), 116.7 (C-5′), 123.3 (C-6′), 103.5 (C-1″), 72.2 (C-2″), 72.1 (C-3″), 73.4 (C-4″), 72.0 (C-5″), 17.7 (C-6″) [27,28].

## 4. Conclusions

**Figure 9:**
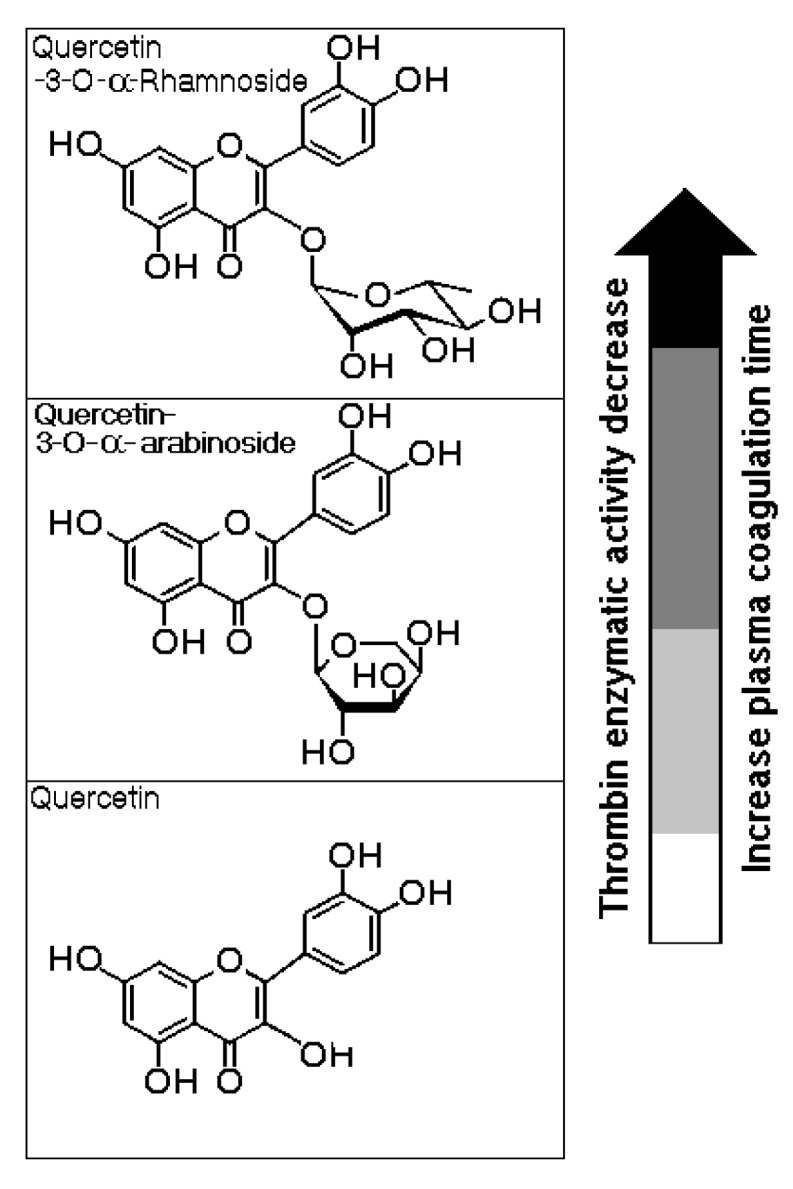
Correlation between the structures of quercetin-3-*O*-rhamnoside (Qn) and quercetin-3-*O*-arabinoside (QAra) and their effects on thrombin and the plasma coagulation time.

As a final consideration, knowledge of the structure–activity relationships between flavonoids such as quercetin or glycosylated quercetin derivatives could enable the design of chemical compounds with higher potencies as thrombin inhibitors. Moreover, these structure-activity relationships could provide information to facilitate the exploitation and utilization of flavonoids as thrombin inhibitors in the treatment of thrombotic diseases. In addition, our results show that glycosylation is extremely important for the inhibitory activities of these compounds against TH. As summarized in Figure 9, the sugar type also has a strong influence on this effect. Finally, mangrove ecosystems have been degraded and have disappeared from some countries. In this article, we show that mangrove plants should be considered important sources of active molecules.
